# Supplementary material for: Chromothripsis during telomere crisis is independent of NHEJ, and consistent with a replicative origin
Source: Genome Res. 2019 May;29(5):737–49. doi: 10.1101/gr.240705.118 (PMC6499312; doi:10.1101/gr.240705.118)
Supplement: Supplemental Material [file supp_gr.240705.118_Supplemental_file_1.zip › contigs/annotated_contigs/DB46/contig.2.DB46_length_351_mean_cov_4.56125356125.docx]

**DB46_length_351_mean_cov_4.56125356125**

```
ATTTCTTCACCGTTGGAAACTTTTCTAAGGGCAAATCAACTCCAGTTGAGTGCAACTTAAACTCTCAGCGTGATGTTTACTGGGTGTCC
 >chr18:55033762-55033992 - E=2e-122
CCTGGTGGGGTAGAGACCTTGGAACCTCAGTGCCTTTCCAGATATGTAGAGCCAAGTGAGCTTACTGGGTGCCTTGTTCTTCCCCTGCT

CCTTTGCAGGCACTGTTTTGTGGGTACCTGTATTTTTTTTTTATTGTGTCAG|AA|AATACCTTATTGAAGAAAAAGGCCACAAACACT
                                                     >chr18:54986760-54986881 + E=2e-61
AGCTTATTCATGATACTATCTCCAGATGACCTTAAAGAGATCCAATAACTCCAAAACCATGAACTTTAGAGGCTCTGATGCAACTA
```
